# PKCγ receptor mediates visceral nociception and hyperalgesia following exposure to PTSD-like stress in the spinal cord of rats

**DOI:** 10.1186/1744-8069-9-35

**Published:** 2013-07-09

**Authors:** Yu-Qin He, Qiang Chen, Lei Ji, Zheng-Guo Wang, Zhi-Hong Bai, Robert L Stephens, Min Yang

**Affiliations:** 1 Department of Gastroenterology, Daping Hospital, Third Military Medical University, 10 Changjiang Branch Road, Daping, Chongqing 400042, China; 2 School of Psychology, Southwest University, Beibei, Chongqing 400715, China; 3 Department of Physiology and Cell Biology, The Ohio State University, 43210, Columbus, Ohio, USA

**Keywords:** Visceral hyperalgesia, Protein kinase C gamma, Post-traumatic stress disorder, Single-prolonged stress, Colorectal distention, Visceromotor response, Spinal cord

## Abstract

**Background:**

Clinical studies indicate that patients with post-traumatic stress disorder (PTSD) frequently share comorbidity with numerous chronic pain conditions. However, the sustained effects of PTSD-like stress over time on visceral nociception and hyperalgesia have been rarely studied, and the underlying mechanisms of stress-induced modulation of visceral hyperalgesia remain elusive. The purpose of this study was to investigate the characterization of visceral nociception and hyperalgesia over time in rats exposed to PTSD-like stress, and to explore the potential role of protein kinase C gamma (PKCγ) in mediating visceral hyperalgesia following exposure to PTSD-like stress.

**Results:**

On day 1, the rats exposed to single-prolonged stress (SPS, an established animal model for PTSD) exhibited an analgesic response and its visceromotor response (VMR) to graded colorectal distention (CRD) at 40 and 60 mmHg was reduced compared with the control group (all *P* < 0.05). On day 6, the VMR returned to the baseline value. However, as early as 7 days after SPS, VMR dramatically increased compared with its baseline value and that in the controls (all *P* < 0.001) and this increase persisted for 28 days, with the peak on day 9. Abdominal withdrawal reflex (AWR) scores were higher in SPS rats than in controls on days 7, 9, 14, 21 and 28 (all *P* < 0.001). Intrathecal administration of GF109203X (an inhibitor of PKC gamma), attenuated the SPS-induced increase in both VMR and AWR scores on days 7, 14, 21 and 28 (all *P* < 0.05). PKCγ protein expression determined by immunofluorescence was reduced in the spinal cord within 3 days after the exposure to SPS (*P* < 0.01), which returned to normal levels between days 4 and 6, and significantly increased from day 7, and this increase was maintained on days 14, 21, and 28 (all *P* < 0.001), with the peak on day 9. In addition, Western blotting showed a consistent trend in the changes of PKCγ protein expression.

**Conclusions:**

The modified SPS alters visceral sensitivity to CRD, and contributes to the maintenance of visceral hyperalgesia, which is associated with enhanced PKCγ expression in the spinal cord. Functional blockade of the PKCγ receptors attenuates SPS-induced visceral hyperalgesia. Thus, the present study identifies a specific molecular mechanism for visceral hyperalgesia which may pave the way for novel therapeutic strategies for PTSD-like conditions.

## Introduction

Post-traumatic stress disorder (PTSD) is a group of symptoms that occur in individuals who have been exposed to life-threatening stressors [1-3]. Epidemiological studies have implicated PTSD-like stress as a trigger of first onset or exacerbation or relapse of symptoms of irritable bowel syndrome (IBS), characterized by hyperalgesia and allodynia [4,5]. Despite the high comorbidity of chronic pain conditions in PTSD patients [6-11], the sustained effects of PTSD-like stress over time on visceral nociception and hyperalgesia have been rarely studied. Furthermore, the molecular mechanisms underlying stress-induced modulation of visceral pain and prolonged visceral hyperalgesia remain unknown. Moreover, limited data on visceral pain processing in this disorder show inconsistent results [9,12,13], highlighting the need for further investigation. Previous studies on PTSD have focused predominantly on the amygdala, medial prefrontal cortex, hippocampus and anterior cingulate of the brain [14-16]. However, there is a growing appreciation that the dorsal horn neurons of the spinal cord are also impacted by the stress, which can last for weeks [17,18]. Thus, spinal sensitization is increasingly accepted as an important component in the maintenance of allodynia and hyperalgesia in various models of chronic pain and stress-induced visceral hypersensitivity [19-22]. The single-prolonged stress (SPS) model, an established animal model for PTSD, mimics some of the physiological and behavioral changes described in PTSD patients, and has been used to examine the therapeutic responses in visceral pain related to the intense stress [9,12,23,24].

The gamma isoform of protein kinase C (PKCγ) is widely distributed throughout the nervous system, particularly in the interneurons of the inner part of lamina II of the dorsal horn, which implies that PKCγ may play a critical role in the nociceptive signaling process [25]. Accordingly, a variety of approaches have been explored to elucidate the potential role of PKCγ as a pain mediator in nociceptive signal transduction [26-29]. It has been demonstrated that PKCγ is involved in many aspects of cellular sensitization, including modulation of channel conductivity, increased trafficking of receptors, and release of excitatory neurotransmitters [30]. PKCγ plays an important role in the sensitization of nociceptive neurons of dorsal horn in certain pain states and thus algesic hypersensitivity in several animal models of visceral pain and visceral injury [9,16,30-32]. PKCγ expression is upregulated under pain conditions resulting from nerve damage or inflammation in animal models [26-29], indicating its involvement in both neuropathic and inflammatory pain. Lower mechanical and thermal hyperalgesia has been observed in PKCγ knock-out mice in response to nerve damage [26,30,33], and spinal blockade of PKCγ reverses the hyperalgesia induced by subcutaneous formalin, pancreatitis and cutaneous capsaicin [34-37]. Although all these data support a role for PKCγ in visceral pain, it is unknown whether PKCγ also contributes to the maintenance of chronic visceral hyperalgesia over time following exposure to PTSD-like stress. We postulate that PKCγ is the potential mechanism through which spinal sensitization may occur in response to PTSD-like stress.

Therefore, the purpose of this study was to investigate the characterization of visceral nociception and hyperalgesia over time in rats exposed to PTSD-like stress, and to explore the potential role of PKCγ in mediating visceral hyperalgesia following exposure to PTSD-like stress. This study showed that SPS altered visceromotor response (VMR) and abdominal withdrawal reflex (AWR) to graded colorectal distention (CRD) and contributed to the development of delayed visceral hyperalgesia, which is accompanied by PKCγ overexpression. GF109203X (an inhibitor of PKCγ) attenuated the VMR and AWR in SPS-exposed rats. Thus, the present study provides direct evidence for the role of PKCγ in SPS-induced visceral hyperalgesia, and may pave the way for novel therapeutic strategies for PTSD-like stress.

## Results

### Alteration of VMR to graded CRD over time following exposure to SPS

**Figure 1:**
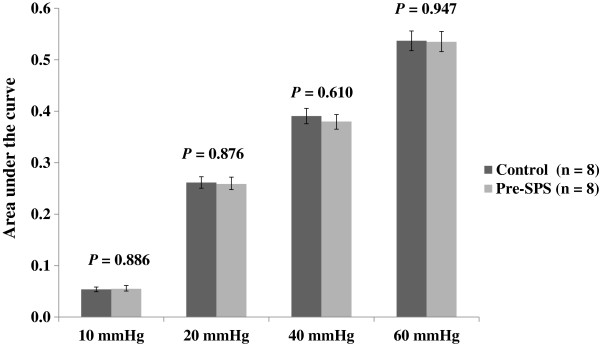
**Baseline visceromotor response to graded colorectal distension in the control rats and those to be exposed to single**-**prolonged stressPre**-**(SPS).**

VMR to CRD was recorded at different time points (baseline, days 1, 6, 7, 10, 14, 21, 28 and 29) after SPS exposure. The baseline VMR to CRD was equivalent between control and pre-SPS groups in a pressure-dependent manner (Figure 1). However, on day 1, SPS-treated rats exhibited an analgesic response, and the VMR to CRD was reduced at both 40 and 60 mmHg compared with control rats (0.301 ± 0.012 *vs*. 0.380 ± 0.014, *P* = 0.013 and 0.417 ± 0.018 *vs*. 0.535 ± 0.020, *P* < 0.001, respectively). On day 6, the VMR returned to the baseline values in SPS rats. However, on day 7, the VMR to CRD was significantly increased at both 40 and 60 mmHg, and the mean area under the curve (AUC) of electromyographic (EMG) signal in SPS rats was significantly increased compared with its baseline value and that in the controls (all *P* < 0.001). There was a significant increase in the VMR to graded intensities of phasic CRD (40 and 60 mmHg) between day 7 and day 28 compared with baseline value in SPS rats and those in the control rats (all *P* < 0.001), with the peak on day 9 after SPS exposure. On day 29, the VMR decreased to a level similar to baseline levels (Table 1).

**Table 1:** **Visceromotor response** (**VMR**) **to phasic ascending colorectal distention** (**CRD**) **over time following exposure to single**-**prolonged stress**

**Group**	**10 mm Hg**	**20 mm Hg**	**40 mm Hg**	**60 mm Hg**
Control (n = 8)	0.055 ± 0.006	0.259 ± 0.013	0.380 ± 0.014	0.535 ± 0.020
Post-1 day (n = 7)	0.053 ± 0.007	0.230 ± 0.012	0.301 ± 0.012^*∇^	0.417 ± 0.018^*∇^
Post-6 days (n = 8)	0.047 ± 0.006	0.263 ± 0.019	0.359 ± 0.012	0.536 ± 0.022
Post-7 days (n = 10)	0.056 ± 0.006	0.284 ± 0.020	0.617 ± 0.022^▲∇^	0.759 ± 0.022^▲∇^
Post-9 days (n = 7)	0.059 ± 0.007	0.329 ± 0.015	0.670 ± 0.028^▲∇^	0.896 ± 0.008^▲∇^
Post-14 days (n = 8)	0.048 ± 0.005	0.254 ± 0.037	0.653 ± 0.020^▲∇^	0.818 ± 0.020^▲∇^
Post-21 days (n = 10)	0.053 ± 0.006	0.291 ± 0.017	0.648 ± 0.021^▲∇^	0.823 ± 0.017^▲∇^
Post-28 days ( n = 9)	0.050 ± 0.005	0.312 ± 0.016	0.623 ± 0.024^▲∇^	0.839 ± 0.026^▲∇^
Post-29 days (n = 9)	0.042 ± 0.003	0.291 ± 0.021	0.450 ± 0.022	0.594 ± 0.025

All data are expressed as the mean ± S.E.M.

VMR was represented by the mean value of the area under the curve of the electromyographic activity during CRD.

* *P* < 0.05 compared with Control.

^▲^*P* < 0.001 compared with Control.

^∇^*P* < 0.05 compared with post-29 days.

At a distension pressure of 60 mmHg, 87% of the SPS-exposed rats developed an increased VMR 7 days following exposure to SPS; 22%, 38% and 27% exhibited an increase in VMR by 1–50%, 51–100%, and > 101% over the baseline, respectively.

### AWR in response to graded CRD over time following exposure to SPS

Visceral pain sensitivity was also determined by measuring the AWR scores in response to graded CRD (10, 20, 40 and 60 mmHg) at various time points (baseline, days 1, 6, 7, 14, 21, 28 and 29 after SPS). The AWR scores were higher in rats exposed to SPS at 40 and 60 mmHg than in control rats on days 7, 9, 14, 21 and 28 (all *P* < 0.001) (Table 2).

**Table 2:** **Abdominal withdrawal reflex score in response to phasic ascending colorectal distention over time following exposure to single**-**prolonged stress**

**Group**	**10 mm Hg**	**20 mm Hg**	**40 mm Hg**	**60 mm Hg**
Control (n = 8)	1.099 ± 0.120	2.050 ± 0.109	2.586 ± 0.134^▲^	2.850 ± 0.132^▲^
Post-1 day (n = 7)	1.117 ± 0.159	2.029 ± 0.157	1.942 ± 0.149^*^&	1.986 ± 0.122^*^&
Post-6 days (n = 8)	1.170 ± 0.113	2.050 ± 0.109	3.125 ± 0.096^*▲^&	3.625 ± 0.206^*▲^&
Post-7 days (n = 10)	1.067 ± 0.072	2.090 ± 0.067	3.190 ± 0.057^*▲^&	3.860 ± 0.134^*▲^&
Post-9 days (n = 7)	1.043 ± 0.100	2.100 ± 0.079	3.186 ± 0.083^*▲^&	3.871 ± 0.161^*▲^&
Post-14 days (n = 8)	1.100 ± 0.101	2.050 ± 0.065	3.162 ± 0.065^*▲^&	3.650 ± 0.093^*▲^&
Post-21 days (n = 10)	1.109 ± 0.081	2.040 ± 0.056	3.170 ± 0.063^*▲^&	3.800 ± 0.089^*▲^&
Post-28 days ( n = 9)	1.078 ± 0.101	2.022 ± 0.061	3.222 ± 0.055^*▲^&	3.522 ± 0.072^*▲^&
Post-29 days (n = 9)	1.080 ± 0.085	2.000 ± 0.058	2.638 ± 0.091^▲^	2.800 ± 0.097^▲^

All data are expressed as the mean ± S.E.M.

* *P* < 0.001 compared with Control.

^▲^*P* < 0.001 compared with post-1 day.

&*P* < 0.001 compared with post-29 days.

### Effects of GF109023X on VMR to CRD following exposure to SPS

**Figure 2:**
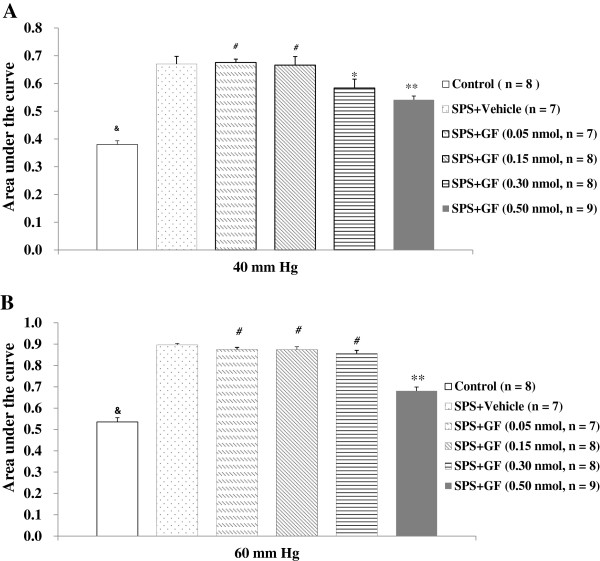
**Effect of GF109203X (GF) at different doses on visceromotor response to phasic colorectal distention at 40 mmHg (A) and 60 mmHg (B) 9 days following exposure to single-prolonged stress (SPS).** All data are expressed as the mean ± S.E.M. * *P* < 0.05, and ** *P* < 0.001, compared with SPS + Vehicle, respectively; &*P* < 0.001, compared with SPS + Vehicle; ^#^*P* < 0.001, compared with SPS + GF (0.5 nmol).

Since the peaking VMR to phasic CRD (40 and 60 mm Hg) was on day 9 after exposure to SPS, we chose this time point to investigate the dose–response effects of GF109023X at doses of 0.05-0.50 nmol/10 μL on VMR to CRD, and determined the effective dose that completely inhibits the response to 60 mmHg CRD, a noxious intensity. When administered intrathecally, GF109203X at low doses ranging from 0.05-0.15 nmol/10 μL did not significantly affect VMR to the graded intensities of phasic CRD. However, at 0.30 nmol/10 μL, GF109023X significantly attenuated VMR to CRD at 40 mmHg in the SPS-treated rats compared with the vehicle group (0.584 ± 0.032 *vs*. 0.670 ± 0.028, *P* = 0.013); no such effect was observed at 60 mmHg. At 0.50 nmol/10 μL, GF109023X completely inhibited the response to CRD at both 40 mmHg (0.541 ± 0.014 *vs*. 0.670 ± 0.028, *P* < 0.001) and 60 mmHg (0.681 ± 0.018 *vs*. 0.896 ± 0.008, *P* < 0.001) in SPS-treated rats compared with the vehicle group and with the low-dose groups (all *P* < 0.001) (Figures 2A &2B, Table 3).

**Table 3:** **Effect of GF109203X** (**GF**) **on visceromotor response to phasic ascending colorectal distention over time following exposure to single**-**prolonged stress** (**SPS**)

**Group**	**10 mm Hg**	**20 mm Hg**	**40 mm Hg**	**60 mm Hg**
Control (n = 8)	0.055 ± 0.006	0.259 ± 0.013	0.380 ± 0.014^$^	0.535 ± 0.020^$^
SPS + Vehicle (n = 7)	0.059 ± 0.007	0.299 ± 0.013	0.670 ± 0.028^#^	0.896 ± 0.008^#^
SPS + GF (0.05 nmol) (n = 7)	0.059 ± 0.004	0.293 ± 0.021	0.676 ± 0.012^#^	0.874 ± 0.010^#^
SPS + GF (0.15 nmol) (n = 8)	0.059 ± 0.004	0.281 ± 0.017	0.666 ± 0.031^#^	0.874 ± 0.014^#^
SPS + GF (0.30 nmol) (n = 8)	0.054 ± 0.004	0.278 ± 0.019	0.584 ± 0.032^*^	0.856 ± 0.015^#^
SPS + GF (0.50 nmol) (n = 9)	0.053 ± 0.003	0.277 ± 0.015	0.541 ± 0.014^**^	0.681 ± 0.018^**^

All data are expressed as the mean ± S.E.M.

* *P* < 0.05, and ** *P* < 0.001, compared with SPS + Vehicle, respectively.

^$^*P* < 0.001, compared with SPS + Vehicle.

^#^*P* < 0.001, compared with SPS + GF (0.50 nmol).

**Figure 3:**
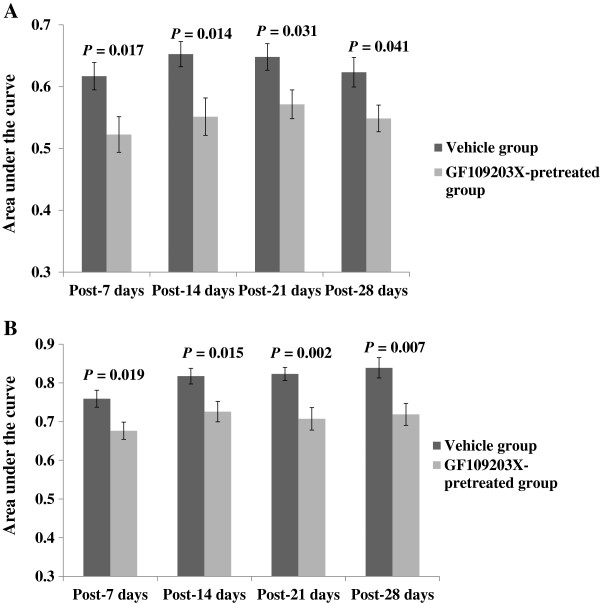
**Effect of GF109203X (GF) at 0.5 nmol/10 μL on visceromotor response to phasic colorectal distention at 40 mmHg (A) and 60 mmHg (B) following exposure to single-prolonged stress (SPS).** All data are expressed as the mean ± S.E.M. (n = 7–10). All *P* < 0.05, compared with the Vehicle group.

In addition, intrathecal administration of 0.50 nmol/10 μL of GF109203X 10 min before CRD abolished visceral hyperalgesia and attenuated SPS-induced increase in VMR to CRD at 40 and 60 mmHg on days 7, 14, 21 and 28 after SPS, when compared with vehicle injection (all *P* < 0.05) (Figures 3A &3B). In control rats, GF109203X had no significant effects on the VMR to CRD.

### Effects of GF109023X on AWR to CRD following exposure to SPS

**Figure 4:**
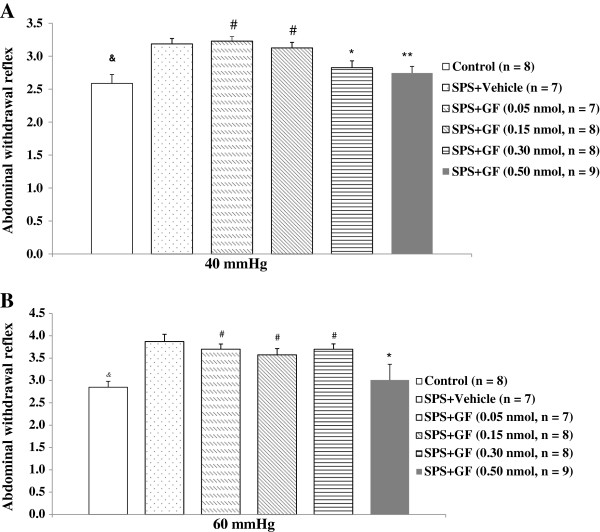
**Effect of GF109203X (GF) at different doses on abdominal withdrawal reflex score in response to phasic colorectal distention at 40 mmHg (A) and 60 mmHg (B) following exposure to single-prolonged stress (SPS).** All data are expressed as the mean ± S.E.M. * *P* < 0.05, and ** *P* < 0.01, compared with SPS + Vehicle; ^#^*P* < 0.05, compared with SPS + GF (0.5 nmol); &*P* < 0.01, compared with SPS + Vehicle.

We also investigated the dose–response effects of GF109023X on AWR to CRD on day 9 after exposure to SPS. At low-doses (0.05-0.15 nmol/10 μL), GF109203X had no significant effects on AWR to the graded intensities of phasic CRD. However, at 0.30 nmol/10 μL, GF109023X significantly attenuated the AWR to CRD at 40 mmHg in SPS-treated rats compared with the vehicle group (2.825 ± 0.103 *vs*. 3.186 ± 0.083, *P* = 0.017); no such effect was observed at 60 mmHg. At 0.50 nmol/10 μL, GF109023X completely attenuated the AWR in response to CRD at both 40 and 60 mmHg, with the AWR scores to CRD being significant decreased in SPS-treated rats when compared with the vehicle group (2.744 ± 0.100 *vs*. 3.186 ± 0.083, *P* = 0.003 for 40 mmHg and 3.011 ± 0.353 *vs*. 3.871 ± 0.161, *P* = 0.005 for 60 mmHg), and with the low-dose groups (all *P* < 0.05) (Table 4 and Figures 4A &4B).

**Table 4:** **Effect of GF109203X** (**GF**) **on abdominal withdrawal reflex score in response to phasic ascending colorectal distention over time following exposure to single**-**prolonged stress** (**SPS**)

**Group**	**10 mm Hg**	**20 mm Hg**	**40 mm Hg**	**60 mm Hg**
Control (n = 8)	1.099 ± 0.120	2.050 ± 0.109	2.588 ± 0.134&	2.850 ± 0.132&
SPS + Vehicle (n = 7)	1.043 ± 0.010	2.100 ± 0.079	3.186 ± 0.083	3.871 ± 0.161
SPS + GF (0.05 nmol) (n = 7)	1.153 ± 0.098	2.071 ± 0.102	3.229 ± 0.068^#^	3.700 ± 0.115^#^
SPS + GF (0.15 nmol) (n = 8)	1.030 ± 0.065	2.088 ± 0.048	3.125 ± 0.084^#^	3.575 ± 0.139^#^
SPS + GF (0.30 nmol) (n = 8)	1.120 ± 0.087	2.038 ± 0.080	2.825 ± 0.103^*^	3.700 ± 0.118^#^
SPS + GF (0.50 nmol) (n = 9)	1.060 ± 0.064	1.967 ± 0.067	2.744 ± 0.100^**^	3.011 ± 0.353&

All data are expressed as the mean ± S.E.M.

* *P* < 0.05, and ** *P* < 0.01, compared with SPS + Vehicle, respectively.

^#^*P* < 0.05, compared with SPS + GF (0.50 nmol).

&*P* < 0.01, compared with SPS + Vehicle.

**Figure 5:**
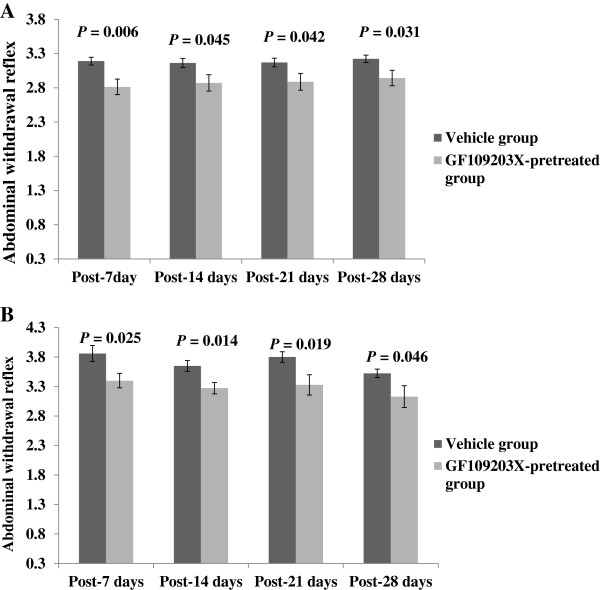
**Effect of GF109203X (GF) at 0.5 nmol/10 μL on abdominal withdrawal reflex score in response to phasic colorectal distention at 40 mmHg (A) and 60 mmHg (B) following exposure to single-prolonged stress (SPS).** All data are expressed as the mean ± S.E.M. (n = 7–10). All *P* < 0.05, compared with the Vehicle group.

In addition, the administration of GF109203X at 0.50 nmol/10 μL caused a significant reduction of AWR to CRD at 40 and 60 mmHg on days 7, 14, 21 and 28 compared with baseline and vehicle injection (all *P* < 0.05) (Figures 5A &5B). In control rats, GF109203X had no significant effects on the AWR to CRD.

### Spinal PKCγ protein expression over time following exposure to SPS as determined by immunofluorescence staining

**Figure 6:**
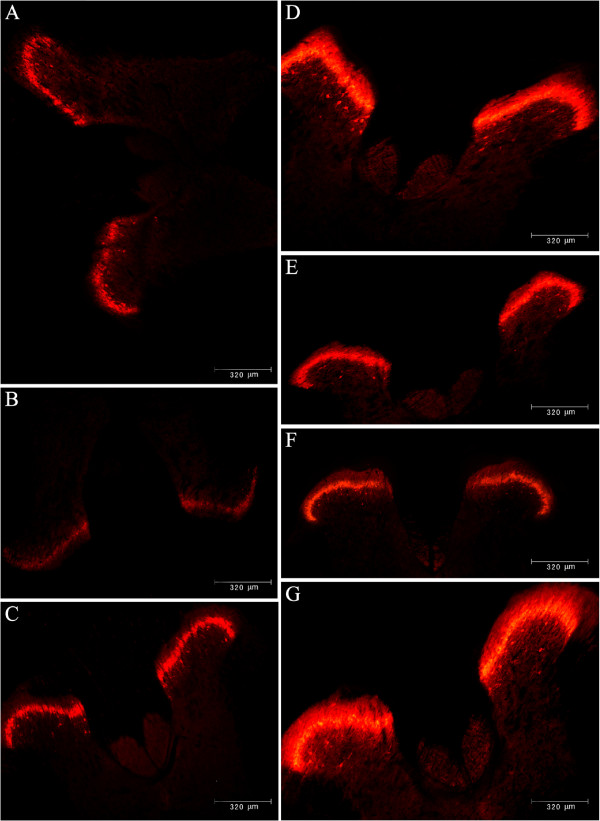
**PKCγ protein expression by immunofluorescence in the spinal level over time following exposure to single**-**prolonged stress.** Representatives illustrating the distribution of PKCγ-immunoreactivity (IR) in the dorsal horn of control rats **(A)**, and those day 1 **(B)**, 7 **(C)**, 14 **(D)** 21 **(E)**, 28 **(F)**, 9 **(G)** after exposure to SPS.

In the dorsal horn of the spinal cord, PKCγ immunostaining formed a dense plexus, consisting of strongly immunoreactive cell bodies and associated dendrites. The most strongly PKCγ-immunoreactive neurons were located in the inner plexus of lamina II (Iii) and ventral to the plexus in lamina III, whereas weakly stained cells were present in the outer part of lamina II (IIo) and in lamina I. By immunofluorescence analysis, SPS-treated rats exhibited a reduced PKCγ expression in the dorsal horn of the spinal cord within 3 days after exposure to SPS compared with baseline (*P* < 0.01, Figures 6A &6B); however, those neuronal stores were replenished between days 4 and 6 after exposure to SPS. SPS significantly increased PKCγ expression in the dorsal horn of the spinal cord as early as on day 7, compared with the control rats, with the average optical density (AOD) of immunoreactivity being 143.44 ± 2.40 *vs*. 108.50 ± 3.66, *P* < 0.001 (Figure 6C). This increase was maintained on days 14 (148.22 ± 4.02), 21 (147.33 ± 3.77), and 28 (149.11 ± 4.68) in SPS-treated rats compared with the control rats (all *P* < 0.001) (Figures 6D-F, Table 5). A similar trend was noted on day 29 after SPS, but this was not statistically significant (*P* = 0.420). The peak expression of PKCγ was reached on day 9 (Figure 6G). In the meantime, there was no marked difference in the PKCγ expression in the SPS exposed rats among different time points (all *P* > 0.05). Negative controls (treated without the primary antibody) had no staining.

**Table 5:** **Average optical density of PKCγ immunofluorescence in the dorsal horn of the spinal cord of rats following exposure to single**-**prolonged stress**

**Group**	**AOD values**
Control (n = 8)	108.50 ± 3.66
Post-1 day (n = 7)	90.57 ± 2.83^*^
Post-6 days (n = 7)	102.57 ± 3.21^∇^
Post-7 days (n = 9)	143.44 ± 2.40^**▲^&
Post-9 days (n = 8)	153.50 ± 3.13^**▲^&
Post-14 days (n = 9)	148.22 ± 4.02^**▲^&
Post-21 days (n = 9)	147.33 ± 3.77^**▲^&
Post-28 days (n = 9)	149.11 ± 4.68^**▲^&
Post-29 days (n = 8)	104.25 ± 3.97^∇^

All data are expressed as the mean ± S.E.M.

*P < 0.01 and **P < 0.001, compared with control group, respectively.

^▲^*P* < 0.001 compared with post-1 days group.

^∇^*P* < 0.05 compared with post-1 days group.

&*P* < 0.05 compared with post-29 days group.

### Spinal PKCγ protein expression over time following exposure to SPS as determined by Western blotting

**Figure 7:**
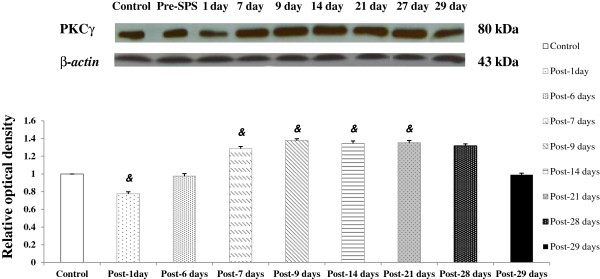
**PKCγ protein expression in the spinal cord over time following exposure to single-prolonged stress as determined by Western blotting.** &*P* < 0.001 compared with the Control.

The PKCγ protein expression was analyzed with Western blotting from the spinal cord homogenates. On day 1, the relative optical density (ROD) of the PKCγ immunoblot bands in the spinal cord was significantly decreased in SPS-exposed rats, compared with the control rats (*P* < 0.001). PKCγ expression was significantly increased on as early as day 7 in SPS-exposed rats, and this increase was also maintained on days 14, 21 and 28, compared with the controls (all *P* < 0.001) (Figure 7 and Table 6).

**Table 6:** **Relative optical density of PKCγ protein expression in the spinal cord of rats following exposure to single**-**prolonged stress as determined by Western blotting**

**Group**	**ROD**
Control (n = 7)	1.000 ± 0.000^▲^
Post-1 day (n = 7)	0.774 ± 0.024^*^&
Post-6 days (n = 6)	0.975 ± 0.028^▲^
Post-7 days (n = 8)	1.284 ± 0.025^*▲^&
Post-9 days (n = 7)	1.376 ± 0.021^*▲^&
Post-14 days (n = 8)	1.345 ± 0.027^*▲^&
Post-21 days (n = 7)	1.351 ± 0.027^*▲^&
Post-28 days (n = 8)	1.316 ± 0.023^*▲^&
Post-29 days (n = 7)	0.990 ± 0.019^▲^

All data are expressed as the mean ± S.E.M.

* *P* < 0.001 compared with control group.

^▲^*P* < 0.001 compared with post-1 days group.

&*P* < 0.001 compared with post-29 days group.

## Discussion

Clinical studies indicate that PTSD patients frequently shares comorbidity with numerous chronic pain conditions. However, only a few studies have examined if subjection to PTSD-like conditions alters visceral nociception and hyperalgesia, which reported decreased or unchanged nociceptive sensitivity [8,9,11]. In addition, the molecular mechanisms underlying stress-induced modulation of visceral hyperalgesia remain elusive. PKCγ as a pain marker has been shown to be important in several animal models of visceral pain, and is thought to play a role in long term hyper-excitability or sensitization in nociceptive neurons of dorsal horn in certain pain states [25-27,29-32]. We hypothesized that PTSD-like stress alters visceral sensitivity and produces visceral hyperalgesia, and spinal activation of PKCγ mediates the development of persistent visceral hyperalgesia following exposure to PTSD-like stress. Therefore, we examined the characterization of visceral nociception and hyperalgesia over time following exposure to PTSD-like stress, and explored the role of PKCγ signaling in the development of stress-induced visceral hyperalgesia at the spinal level and provided novel insights into its underlying molecular mechanisms of stress-related pain modulation.

In present study, we used a previously validated rat model of modified SPS, an established animal model for PTSD [23,24]. The inescapable electric foot shock, which is often employed to produce fear or anxiety and considered a nociceptive stimulus, was added to the conventional SPS procedures, and reported to significantly enhance conditioned and sensitize fear responses [24]. This model mimics some of the physiological, behavioral changes, hypothetical pathogenesis and clinical pain features described in PTSD patients that includes enhanced negative feedback to the hypothalamic-pituitary-adrenal axis, anxiety-like behavior, cognitive impairments and memory dysfunction [23,24,38]. This model is also responsive to treatments showing some efficacy in alleviating visceral pain in IBS patients and therefore can predict treatment responses to specific drugs or nonpharmacological interventions in humans [9,12].

Previous animal studies have demonstrated that acute and chronic stress is associated with the development of allodynia, hyperalgesia or unaltered nociceptive sensitivity [17,18,39,40]. For example, 10–20 min forced swimming daily for 3 days induces hyperalgesia to thermal and chemical stimuli 8 to 9 days after the last swim session [17]. Exposure to water avoidance stress (WAS) for 1 h leads to a delayed visceral hyperalgesia to CRD, appearing 24 h after the end of the stress, and 10-day homotypic water avoidance stress induces visceral hypersensitivity that lasts for about 40 days [18]. Interestingly, exposure to a stressor stronger than WAS was found to induce an immediate hyperalgesia to CRD [41]. Moreover, the stress-related experimental models of IBS have demonstrated a lowered pain threshold and hyperalgesia to CRD [12,18,42-44]. In addition, female rats appear to show a different pattern of sensitized behavioral responsiveness to the same challenge, indicating sex-related alterations in the neuronal substrates involved in the responsiveness [12,41,44].

The present study demonstrated that there was a significant effect of SPS on VMR and AWR to distention, indicating that changes in visceral nociception and hyperalgesia differed over time between control and SPS rats. SPS-treated rats exhibited a temporary visceral analgesia within the initial 3 days following SPS exposure. Then, it was replenished on days 4 and 5, and returned to the baseline on day 6. SPS-induced analgesia on day 1 may be mediated by activation of the descending inhibitory pain pathway [45-47]. Pharmacological and neurochemical studies have demonstrated involvement of a large number of neurotransmitters and neuropeptides, such as endogenous opioid, monoamine, cannabinoid, g-aminobutyric acid and glutamate systems [45-47]. In addition, the present study showed down-regulation of PKCγ on day 1 after SPS, which may be another potential mechanism for stress-induced analgesia. These findings may enhance our understanding of the fundamental pathophysiology of SPS-induced visceral analgesia; however, further studies are required to determine whether the PKCγ signaling pathway can be a new therapeutic target for the treatment of stress-related functional gastrointestinal disorders.

In the present study, both VMR and AWR to CRD dramatically increased 7 days after initiation of SPS and the increased nociceptive responses were maintained for up to 28 days. However, visceral sensitivity did not differ within the control group over the 28 day period, indicating that the rats did not become sensitized to repeated CRD assessments over time. The development of the peripheral and central sensitization may be important in mediating stress-induced visceral hypersensitivity to CRD, in which maladaptive neuroplastic changes lead to persistently increased perception and response to noxious, or non-noxious stimuli [9,48,49]. Moreover, it has been reported that once peripheral and central sensitization has developed, it can in turn activate the release of spinal cord mediators such as acid-sensing ion channel 1a, neurokinin-1, and growth factors such as nerve growth factor or brain-derived neurotrophic factor and phosphorylation of extracellular signal-regulated kinases 1 and 2 (ERK1/2) as a result of stress exposure [22,50-52]. In the present study, PKCγ was up-regulated on days 7, 14, 21 and 28 after SPS in the spinal cord, indicating that it may also mediate the development of visceral hyperalgesia. Such alterations in the processing of visceral nociception are all considered as possible mechanisms of chronic visceral hyperalgesia following exposure to PTSD-like conditions.

The present study demonstrated that, concurrent with sustained visceral hyperalgesia, PKCγ protein expression in the dorsal horn neurons of the spinal cord was dramatically increased as early as 7 days after initiation of SPS and sustained for at least another three weeks. This finding indicates that PKCγ protein expression is consistently upregulated and thus PKCγ in the spinal cord is an important intracellular modulator that boosts neuronal activity in algesic and nociceptive signaling pathways. It is conceivable that PKCγ release and biosynthesis are accelerated following exposure to PTSD-like conditions, and the activation of PKCγ may produce an increased visceral nociceptive sensitivity. Although whether elevated PKCγ is a cause of the increased visceral nociceptive sensitivity needs further investigation, increased PKCγ expression at 28 day after initiation of SPS may reflect the broad spectrum of its roles in the development and maintenance of visceral hyperalgesia during PTSD. It is also implied the activation of PKCγ receptors contributes to the development of visceral pain hypersensitivity and hyperalgesia following exposure to PTSD-like stress. Therefore, the present study adds PKCγ to the list of key nociceptive molecules that participate in hypersensitivity in this model and underscores the fact that such visceral sensitization is accompanied by long lasting plasticity of sensory neurons in a PTSD-like stress state.

A previous study demonstrated that GF109203X, a PKCγ inhibitor, at 0.5 nmol/10 μL, a dose several fold higher than the ED50 [30], achieved a significant attenuation of muscle-induced mechanical hyperalgesia. In the present study, visceral nociceptive responses to CRD following exposure to PTSD-like stress was blocked by GF109203X at the spinal level, suggesting that PKCγ inhibitors may be neuroprotective in disorders with dysregulated PKCγ signaling following PTSD-like stress, and thus, supporting that spinal PKCγ activation plays a functional role in the development of visceral hypersensitivity, and enhanced responsiveness after SPS exposure is dependent on PKCγ activation. However, GF109203X had no significant effect on the VMR and AWR scores in control rats, suggesting that this agent did not act as a non-specific analgesic and the role of the PKCγ pathway in signaling colonic distention may not be as important in health as in the sensitized state.

There are several possibilities that could explain how PKCγ activation exerts its effects. First, this protein kinase is able to directly activate ERK1/2 members of the mitogen-activated protein kinases family, p38 and SAP/c-jun terminal kinase, which are involved in pain sensitization and several injury-activated pathways [53-55]. Second, spinal activation of PKCγ involves translocation from the cytosol to binding domains at cell membranes of dorsal horn neurons, increases release of glutamate in the spinal cord, and sensitizes the spinothalamic tract and other dorsal horn neurons, and formalin-induced release of glutamate is prevented by blockade of PKCγ [30,55-57]. Thus, PKCγ produces increased release of glutamate resulting in continued activation of glutamate receptors. Furthermore, spinal activation of PKCγ enhances responses of dorsal horn neurons to N-methyl-D-aspartate (NMDA) and alpha-amino-3-hydroxy-5-methylisoxazole-4-propionic acid agonists, which are involved in NMDA receptor-mediated mechanisms of visceral hyperalgesia. Also, spinal activation of PKCγ increases phosphorylation of the NMDA receptor subunit, NR1, and glutamate receptor subunit, GlurR1, which could result in an increased channel conductance and increase number of its receptors available in the membrane synaptically, resulting in increased excitation of the nociceptive spinal neurons [58-60]. Third, PKCγ decreases the effects of inhibitory neurotransmitters on spinothalamic tract neurons, which is manifested as an increased excitation and the increased PKCγ activity reduces normal inhibition within the spinal cord [61,62]. Therefore, this would result in increased excitability of neurons that is manifested as increased VMR and AWR scores to noxious stimuli.

## Conclusions

The present study indicates that SPS alters visceral sensitivity to CRD, and contributes to the development and maintenance of delayed visceral hyperalgesia, which is associated with an enhanced PKCγ expression in the dorsal horn of the spinal cord. Functional blockade of PKCγ receptors attenuates SPS- induced visceral hyperalgesia. These data indicate that the large enhancement of PKCγ expression and function may contribute to the development and maintenance of visceral hyperalgesia. The present study provides direct evidence for a role of PKCγ in SPS- induced visceral pain, and thus may identify a specific molecular mechanism for visceral hyperalgesia which may pave the way for novel therapeutic strategies for PTSD-like conditions.

## Methods

### Animals

Experiments were performed on 8-week-old female Sprague–Dawley rats (220–300 g). The animals were housed under controlled conditions (21-25°C, 12/12 h light/dark cycle) with availability to standard rat chow and water *ad libitum*. Prior to the experiments, the animals were fasted for 18–24 h with free access to water.

The study protocol with care and handling of these animals was approved by the Institutional Animal Care and Use Committee at the Third Military Medical University. Experiments were performed were in accordance with the Guidelines of the International Association for the Study of Pain. Animals were allowed to acclimate for at least 7 days before experiments.

### Intrathecal catheter implantation

Each of the rats was implanted with a chronic indwelling intrathecal catheter for drug or vehicle delivery. Briefly, animals were placed under general anesthesia using isoflurane inhalation (3.0%). A 23 G needle was inserted into the intrathecal space between L5 and L6 until a tail flick was elicited confirming intrathecal placement. A catheter was considered correctly placed if there was loss of sensory and motor function after injection of lidocaine. A gentamicin sulfate-flushed polyethylene (PE-10) tube was then inserted 4 cm deep so the tip was located for spinal lumbar enlargement. Once the catheter was secured to the fascia, the PE-10 tube was threaded out between the shoulder blades, and the incision was closed with wound clips. Rats were not tested for at least 7 days after surgery. Animals demonstrating motor dysfunction or dehydration immediately following surgery or at any point thereafter were euthanized.

### Drug administration

Drugs were administered to the animals through the intrathecal catheter. A 30 G drug delivery needle attached to a PE-50 tube was affixed to the end of a 50 μL syringe. Drugs were delivered as a 10 μl bolus. For the behavioral experiment, GF109203X (Calbiochem, San Diego, CA), a specific PKC inhibitor, was dissolved in 10% dimethyl sulfoxide (DMSO). Then, GF109203X at different concentrations (0.05-0.50 nmol/10 μL) or vehicle (DMSO) was injected within 2 min directly into the lumbar spinal cord *via* the indwelling intrathecal catheter, and 10 min later, the number of AWR and EMG responses to CRD was measured as described below. SPS exposed rats were treated with one of four different doses of GF109203X alone (0.05, 0.15, 0.30 and 0.50 nmol/10 μL), and the dose–response effects of GF109023X were determined and the effective dose, which was defined as the dose that completely inhibits the response to 60 mmHg CRD, a noxious intensity, was identified for further experiments.

### Modified single-prolonged stress for post-traumatic stress disorder

The detailed SPS procedure was performed as previously described [23,24] with modification. Briefly, animals were restricted in a disposable plastic holder (7 cm diameter, 21 cm in length) for 2 h, and then individually placed in a clear acrylic cylinder (20 cm diameter) filled to two thirds (35 cm) of its height with water (24°C) and forced to swim for 20 min, and following 15 min recuperation, exposed to inhalation of anesthetic isoflurane until the loss of consciousness. When they recovered (approximately 30 min), the electric foot shock (1 mA for 4 s) was delivered *via* metal grids installed in the bottom of the chamber.

### Implantation of EMG electrodes

Currently, the “gold standard” for assessing visceral nociceptive response is recording abdominal smooth muscle contractions, as a proxy to the VMR to CRD in conscious animals. In brief, rats were deeply anesthetized with sodium pentobarbital (45 mg/kg) administered intraperitoneally. Electrodes (Teflon-coated stainless steel wire, Cooner Wire, CA, USA) were stitched into the external oblique musculature, just superior to the inguinal ligament, for EMG recordings as previously described [63]. The electrode leads were then tunneled subcutaneously and externalized laterally through the skin of the abdomen and neck for future access. The incisions were closed with wound clips. Wounds were tested for tenderness to ensure complete recovery from surgery before behavioral testing as described below.

### Behavioral Testing for Nocifensive Responses

#### Visceromotor responses

Visceral sensitivity was measured by grading behavioral response of rats to phasic CRD as previously described [63-65] before initiation of SPS (on day 0, as baseline) and at various time points (on days 1, 6, 7, 9, 14, 21, 28 and 29) in rats exposed to SPS. Briefly, after an overnight fast, the rats were anesthetized using isoflurane inhalation (3.0%). A flexible latex balloon (5 cm) attached to a tygon tube was inserted 8 cm into the descending colon and rectum *via* the anus and held in place by taping the tubing to the base of the tail. After the rat regained consciousness, the rat was allowed to adapt for 30 min prior to CRD. Attachment of a strain gauge force transducer to the abdominal oblique muscle allowed direct monitoring of the muscle contractile activity, and CRD was performed by rapidly inflating the balloon to a constant pressure with a pressure control device (Micro-1401, CED, UK). The distension protocol consisted of a series of phasic CRD to constant pressures of 10, 20, 40, and 60 mmHg, for 20 seconds followed by a 4 min interstimulus interval [64]. To examine the pressure-response relationship, the EMG activity was rectified, and the increase in the AUC of EMG amplitude (over baseline) was recorded as previously described [65].

#### Abdominal withdrawal reflex

Behavioral response to CRD was also measured by visual observation of the AWR, as previously described [13]. The AWR were scored as follows: 0, no behavioral response to CRD; 1, brief head movement followed by immobility; 2, contraction of abdominal muscles; 3, lifting of abdomen; and 4, body arching and lifting of pelvic structures. During testing, the observers were completely blinded to the training conditions.

### Western blotting

Animals were anesthetized with 4% isoflurane and decapitated, and the lumbosacral spinal cord segment was dissected. The tissue, weighing 20 mg, was homogenized in 100 μL of lysis buffer containing 50 mM Tris–HCl pH 8.0, 150 mM NaCl, 1 mM ethylenediamine-N,N,N’,N’-tetraacetic acid (EDTA), 0.5% Triton X-100 and a complete protease inhibitor. The homogenate was incubated on ice for 30 min and then the suspension was sonicated on ice using three 10-second bursts at high intensity with a 10-second cooling period between each burst. The samples were centrifuged at 13000 rpm for 15 min at 4°C and the supernatant was collected and stored at −80°C. The protein concentrations were determined by the BCA Protein Assay Kit as described by the manufacturer. Each protein sample (60 μg) was loaded in 8% sodium dodecyl sulfate-polyacrylamide gel (Bio-Rad), by electrophoresis and then transferred to polyvynilidene fluoride membranes. The membranes were blocked with 5% non-fat dry milk in Tris-buffered saline with 0.5% Tween-20 (TBST) buffer at room temperature for 1 h and incubated with the primary antibody (PKCγ, at 1:1000, Abcam) overnight at 4°C. After washing in TBST, the membranes were incubated with the secondary antibody [horseradish peroxidase (HRP)-conjugated goat anti-rabbit IgG] in 3% milk-TBST (1:3,000 dilution) for 2 h at room temperature. After washing with TBST three times, the HRP-antibody signal was detected by the electrochemiluminescence (ECL) kit from Amersham (GE Healthcare, Piscataway, NJ, USA), followed by exposure to Kodak X-ray film. The membranes were subsequently stripped and re-probed for anti-β-actin antibody (1: 1000, Santa Cruz). Films were scanned and the intensity of PKCγ immunoreactive bands was quantified using Bio-Rad Quantity One software and normalized relative to the intensity of the β-actin immunoreactive band.

### Immunofluorescence staining

For PKCγ immunofluorescence staining, animals were anesthetized with sodium pentobarbital (45 mg/kg) administered intraperitoneally and perfused transcardially with 150 mL phosphate-buffered saline (PBS) followed by 400 mL ice-cold 4% paraformaldehyde (PFA) in phosphate buffer (PB), pH 7.4. Spinal cords were removed and postfixed for 4 h in PFA and transferred to 30% sucrose overnight in PB for cryoprotection, and then cut using a cryostat at 30 μm in thickness. The immunostainings were performed using the ‘free floating’ technique for the spinal cord. Briefly, the sections were blocked for 1 h in blocking buffer (10% donkey serum) at room temperature, and then anti-PKCγ antibody (diluted 1:1000, Abcam) was added overnight at room temperature. After washing three times with TBST at room temperature, the slides were incubated with the following secondary antibodies (Cy3, Invitrogen, diluted 1:200) for 1 h at room temperature. Images were viewed and captured using a BX50 Olympus microscope (Center Valley, PA), using Image-Pro Plus software 6.0 (USA) to analyze the immunofluorescence staining.

### Data analysis

Statistical analyses of the data were performed on the computer using the software PASW 17.0 (Chicago, Illinois, USA). All numerical data were expressed as the mean ± standard error of mean (S.E.M.) and statistical significance was determined by the student t-test or analysis of variance (ANOVA) test, followed by the least significant difference multiple comparison *post hoc* test. *P* values of less than 0.05 were considered statistically significant.

## Abbreviations

AOD: Average optical density; AUC: Area under the curve; AWR: Abdominal withdrawal reflex; CRD: Colorectal distention; DMSO: Dimethyl sulfoxide; EMG: Electromyographic; HRP: Horseradish peroxidase; IBS: Irritable bowel syndrome; PB: Phosphate buffer; PBS: Phosphate- buffered saline; PFA: Paraformaldehyde; PKCγ: Protein kinase C gamma; PTSD: Post-traumatic stress disorder; VMR: Visceromotor response; ROD: Relative optical density; SPS: Single-prolonged stress; WAS: Water avoidance stress.

## Competing interests

The authors declare that they have no financial or nonfinancial competing interests.
